# Editorial: The role of gut microbiota in animal gastrointestinal diseases

**DOI:** 10.3389/fcimb.2025.1554277

**Published:** 2025-01-29

**Authors:** Aoyun Li, Faisal Ayub Kiani, Jianzhao Liao, Fang Liu, Yung-Fu Chang

**Affiliations:** ^1^ College of Veterinary Medicine, Henan Agricultural University, Zhengzhou, China; ^2^ Department of Clinical Sciences, Faculty of Veterinary Sciences, Bahauddin Zakariya University, Multan, Pakistan; ^3^ College of Veterinary Medicine, South China Agricultural University, Guangzhou, China; ^4^ Department of Population Medicine and Diagnostic Sciences, College of Veterinary Medicine, Cornell University, Ithaca, NY, United States

**Keywords:** gut, microbiota, animal, gastrointestinal, disease

The gut microbiota is a complex micro-ecological system that harbors a diverse range of bacteria, fungi, and viruses (Li et al., 2023; Perez, 2021). Recently, it has received significant attention due to its crucial role in host health and physiological functions. Numerous studies have demonstrated that the gut microbiota is closely associated with the host’s nutrient metabolism, digestion, absorption, and the maturation of the immune system (Ansaldo et al., 2019; Wang et al., 2024). Furthermore, research has highlighted its vigorous contributions to intestinal barrier integrity, bone development, epithelial cell differentiation, disease prevention and management (Yuan et al., 2024). As an essential biochemical convertor, the gut microbiota can utilize the food and nutrients ingested by the host to produce metabolites that benefit the host’s health, including short-chain fatty acids, antimicrobial peptides, and vitamins (Liu et al., 2019; Marra et al., 2021). These metabolites are critical for maintaining host health and support various physiological functions. However, the gut microbiota is sensitive to alterations induced by a range of external factors. For instance, age, gender, and species are important internal factors that influence the composition and structure of the gut microbiota (Lee et al., 2023). Additionally, external factors such as heavy metals, antibiotics, and pesticides can markedly disrupt the gut microbiota composition, leading to dysbiosis (Kaur and Rawal, 2023; Li et al, 2024). Research has indicated that dysbiosis is closely associated with the development of various diseases, particularly intestinal disorders such as diarrhea, colitis, and colorectal cancer (Wang et al., 2023). Moreover, dysbiosis of the gut microbiota in the intestine, releases metabolites from certain pathogens, such as lipopolysaccharides which can breach the intestinal barrier and jeopardize host health, contributing to the onset of various diseases (Shen et al., 2021). Therefore, maintaining the stability of the gut microbiota is essential for host health. Given the significant role of the gut microbiota in gastrointestinal diseases, an increasing number of studies are dedicated to exploring the potential relationships between the gut microbiota and the gastrointestinal tract. This Research Topic includes some articles covering all the above aspects.

Rumen acidosis is one of the most prevalent gastrointestinal diseases affecting beef cattle, significantly threatening their health and growth performance, and posing a substantial risk to the beef cattle industry. Despite its importance, relatively few studies examine the potential relationship between rumen acidosis and gut microbiota. Wu et al. conducted a high-throughput sequencing study involving 8 healthy calves and 8 calves diagnosed with rumen acidosis, revealing that rumen acidosis can induce alterations in the composition and diversity of the gut microbiota in calves. Specifically, rumen acidosis was found to affect 70 bacterial genera, with 47 exhibiting increased abundance and 23 showing decreased abundance. Notably, the levels of certain beneficial bacteria, such as Prevotella, Succinivibrio, and Succinivibrionaceae, decreased significantly. These substantial changes in intestinal composition and abundance may serve as critical driving factors for the development of rumen acidosis.

Yaks are an indigenous breed inhabiting the Qinghai-Tibet Plateau, exhibiting strong adaptability to the high-altitude hypoxic environment. Additionally, yaks serve as a vital means of transportation for the residents of the Qinghai-Tibet Plateau and provide milk, meat products, and leather. However, yaks are susceptible to diarrhea caused by *Escherichia coli*, which can lead to significant economic losses and health issues. Diarrhea is one of the primary causes of reduced productivity and mortality in ruminants and is considered a major factor impeding the development of animal husbandry in various countries. Early surveys indicated that diarrhea affects nearly all ruminants, particularly in newborn goats, sheep, cattle, and yaks, whose gastrointestinal tracts are not yet fully developed, resulting in the death of approximately half of these animals. The gut microbiota of ruminants contains a diverse array of beneficial microorganisms, including lactic acid bacteria, bifidobacteria, and lactococci. These probiotics have demonstrated a significant role in managing gastrointestinal diseases in the ruminants. Zhang et al. found that lactic acid bacteria can reduce the translocation rate of pathogens and enhance the intestinal barrier, thereby alleviating diseases caused by *E. coli* in yaks. Consequently, lactic acid bacteria may serve as a promising therapeutic option for treating *E. coli* infections in yaks.

Factors such as transportation, vaccinations, and heat stress can negatively impact calves, resulting in decreased immunity and growth performance, as well as increased morbidity and mortality. The gut microbiota plays a crucial role in host disease resistance, immune system maturation, and metabolism. Furthermore, the gut microbiota is closely associated with host digestion and feed conversion rates. Therefore, enhancing and maintaining the gut microbiota of calves is essential for their health and growth. Yang et al. investigated the effects of supplementation with Shen Qi Bu Qi Powder (SQBQP) on serum biochemistry, antioxidants, gastrointestinal flora, and metabolism in calves. The results indicated that SQBQP supplementation enhanced the growth performance, antioxidant capacity, and digestive enzyme contents in calves. Additionally, SQBQP supplementation improved the gut microbiota and metabolism of calves. This study demonstrates that in light of ongoing demands to reduce antibiotic use, the development of Chinese veterinary compounds aimed at improving the gut microbiota structure of calves is vital for their health and growth.

In recent years, the advancement of science and technology, coupled with the faster turnover of electronic products, has led to explosive growth in industrial production, significantly enhancing people’s quality of life. However, a substantial portion of industrial heavy metal products remains ineffectively recyclable, posing serious threats to the surrounding environment as well as the health of both animals and plants. Furthermore, heavy metals can accumulate in plants and subsequently transfer to other animals and humans through the food chain, presenting a considerable risk to public health and food safety. The intestine serves as the primary pathway for heavy metals to enter the host, indicating that both the intestine and gut microbiota are inevitably affected by these metals. Yang et al. discovered that zinc can induce kidney and intestinal damage, disrupt intestinal barrier function, and cause imbalances in gut microbiota in piglets. Additionally, this study demonstrated that disorders in gut microbiota, which are central to the ‘gut-kidney’ axis, play a significant role in promoting zinc-induced nephrotoxicity. However, supplementation with hesperidin has been shown to mitigate the adverse effects of zinc on piglet health by modulating gut microbiota.
